# Electromagnetic Field Seems to Not Influence Transcription via CTCT Motif in Three Plant Promoters

**DOI:** 10.3389/fpls.2017.00178

**Published:** 2017-03-07

**Authors:** Dariusz Sztafrowski, Anna Aksamit-Stachurska, Kamil Kostyn, Paweł Mackiewicz, Marcin Łukaszewicz

**Affiliations:** ^1^Faculty of Electrical Engineering, Wrocław University of Science and TechnologyWrocław, Poland; ^2^Faculty of Biotechnology, University of WrocławWrocław, Poland

**Keywords:** *Arabidopsis thaliana*, electromagnetic field responsive element, CTCT, *Solanum tuberosum*, 50 Hz magnetic field

## Abstract

It was proposed that magnetic fields (MFs) can influence gene transcription via CTCT motif located in human HSP70 promoter. To check the universality of this mechanism, we estimated the potential role of this motif on plant gene transcription in response to MFs using both bioinformatics and experimental studies. We searched potential promoter sequences (1000 bp upstream) in the potato *Solanum tuberosum* and thale cress *Arabidopsis thaliana* genomes for the CTCT sequence. The motif was found, on average, 3.6 and 4.3 times per promoter (148,487 and 134,361 motifs in total) in these two species, respectively; however, the CTCT sequences were not randomly distributed in the promoter regions but were preferentially located near the transcription initiation site and were closely packed. The closer these CTCT sequences to the transcription initiation site, the smaller distance between them in both plants. One can assume that genes with many CTCT motifs in their promoter regions can be potentially regulated by MFs. To check this assumption, we tested the influence of MFs on gene expression in a transgenic potato with three promoters (16R, 20R, and 5UGT) containing from 3 to 12 CTCT sequences and starting expression of β-glucuronidase as a reported gene. The potatoes were exposed to a 50 Hz 60–70 A/m MF for 30 min and the reporter gene activity was measured for up to 24 h. Although other factors induced the reporter gene activity, the MF did not. It implies the CTCT motif does not mediate in response to MF in the tested plant promoters.

## Introduction

During the life cycle organisms are continually exposed to various external stimuli, which requires adequate responses to maintain homeostasis; this process is often called a stress response. The stress factors cause changes in gene expression resulting in adaptive responses at the proteome (synthesis of relevant proteins) or metabolome levels (production of appropriate metabolites, e.g., antioxidants) (Łukaszewicz and Szopa, 2005).

One of such factors could be extremely low-frequency magnetic fields (ELF-MFs), which influence living organisms are supported by the increasing number of evidences. However, the failure to produce repeatable effects (Heredia-Rojas et al., 2010; Buchachenko, 2016) has made this study difficult and the subject questionable (Blank and Goodman, 2011a; Foster, 2011). Despite many studies, the mechanisms of MF influence are still at the stage of hypotheses rather than well-documented scientific models (Zaporozhan and Ponomarenko, 2010; D’Angelo et al., 2015). Among the various proposed mechanisms, the influence of ELF-MFs on DNA via gene expression is a challenge to test. The interaction could be direct, e.g., DNA can act as fractal antennae (Blank and Goodman, 2011b), or indirect, e.g., free radicals, the circadian clock, or calcium-related pathways can participate in the response (Sztafrowski et al., 2011; Manzella et al., 2015). The influence of ELF-MFs on gene transcription could be mediated by specific sequences, which were found in promoter regions in animals. Indeed, a hypothesis has been proposed that CTCT sequences might act as electromagnetic field response elements (EMREs) in the human HSP70 promoter (Lin et al., 2001). However, it was criticized by other researches (e.g., Alfieri et al., 2006).

Several regulatory mechanisms are similar in animals and plants, especially general stress responses to factors like heat shock or heavy metals (Aksamit et al., 2005). Some data indicate that plants, like animals, perceive and respond to varying MFs by altering their gene expression and phenotypes (Maffei, 2014). However, the influence of MFs on CTCT was studied only in animals and never in plants. It would be interesting to check if these motifs are universal and also mediate responses to ELF-MFs in plants. Therefore, to further examine the hypothesis about CTCT acting as EMRE motifs, we selected a plant model. In particular, we tested if the CTCT motif can regulate gene transcription in response to MF stress in plants. For this purpose, putative promoter regions of all annotated protein-coding nuclear genes from *Solanum tuberosum* and *Arabidopsis thaliana* were analyzed *in silico*. Subsequently, this hypothesis was experimentally evaluated using three promoters that contained CTCT motifs. We selected 16R, 20R and 5UGT promoters, which are involved in flavonoid biosynthesis regulation and responses to free radical stress. The free radicals influence the regulation of genes related to the flavonoid biosynthetic pathway, such as glucose transferase (Lorenc-Kukuła et al., 2004; Korobczak et al., 2005), or regulatory genes encoding 14-3-3 proteins (Szopa et al., 2003a,b; Łukaszewicz et al., 2004). To study the MFs influence, we measured β-glucuronidase (GUS) activity driven by the promoters in a transgenic potato with and without exposure to a 60–70 A/m MF.

## Materials and Methods

### Plant Material and Bacterial Strains

To transform the potato plants (*S. tuberosum L.* cv. *Desiree*) obtained from Saatzucht Fritz Lange KG (Bad Schwartau, Germany), three promoters were used: 20R (EMBL/GenBank database acc. no. AY518222), 5UGT (EMBL/GenBank database acc. no. AY033489), and 16R (EMBL/GenBank database acc. no. AY070220). Each promoter regulated transcription of the reporter gene *uidA* (coding for β-glucuronidase). For plants transformation binary vector pBI101 (Clontech, Mountain View, CA, USA) and *Agrobacterium tumefaciens* strain C58C1 were used. Plants were grown in tissue culture under 16-h light (23 mmol/s/m^2^) – 8-h dark regime in MS medium (Murashige and Skoog, 1962) containing 0.8% sucrose. Plants in the greenhouse were cultivated in soil under 16-h light (in 22°C temperature) – 8-h dark (in 15°C) regime. Plants were grown in individual pots and watered daily.

### Fluorometric GUS Assay

Transcriptional activity of the tested promoters in transgenic plants was measured by GUS reporter gene activity (Łukaszewicz et al., 1998). Briefly, samples were extracted with 50 mM Tris buffer (pH 8.0) containing 10 mM β-mercaptoethanol and 10 mM EDTA, and centrifuged for 10 min at 13 000 rpm. Aliquots of the supernatant were used for an enzyme assay, with 4-methylumbelliferyl β-D-glucuronide as a substrate (Jefferson et al., 1987), and for protein determination with the Bradford reagent (Bradford, 1976). The reaction product, 4-methylumbelliferone, was measured fluorometrically (SFM 25 Fluorescence Spectrophotometer, Kontron Instrument, Hamburg, Germany).

Bioinformatic analyses of tested promoters 16R, 20R and 5UGT identified several regulatory motifs, which could be recognized by various transcription regulation factors (Szopa et al., 2003b; Lorenc-Kukuła et al., 2004; Aksamit et al., 2005). Based on these results, several putative regulatory factors were experimentally tested for each promoter. In this study, we used the following factors inducing the highest and the fastest promoter expression as positive controls: IAA for 16R promoter (Szopa et al., 2003b), ZnSO_4_ for 20R promoter (Aksamit et al., 2005) and ABA for 5UGT promoter (Lorenc-Kukuła et al., 2004). GUS activity was measured in leaves (about 8–12 plastochrons old) incubated on the MS medium supplemented with 2.5% sucrose and 100 μM of the inducing factors.

### Western Blot Analysis

An assessment of the GT protein level was conducted by means of western blot analysis using the rabbit anti-GT IgG and *Solanum sogarandinum* plants. Briefly, the solubilized protein was run on 12% SDS-polyacrylamide gels and blotted electrophoretically onto nitrocellulose membranes (Schleicher and Schuell, Dassel, Germany). After the transfer, the membrane was sequentially incubated with a blocking buffer (5% dry milk) and then with antibodies directed against the GT protein (1:500 dilution). Alkaline phosphatase-conjugated goat anti-rabbit IgG served as the second antibody and was used at the dilution of 1:1500.

### Polymerase Chain Reaction (PCR)

Pooled samples from at least three samples were used for the total RNA extraction. cDNA was synthesized from 5 μg of the total RNA using High Capacity cDNA reverse transcription kit (Applied Biosystems, Poland). cDNA was added to 5 μl of SYBR Green PCR mix (A&A Biotechnology, Gdynia, Poland) and 0.5 μl of each primer (0.5 μm) in triplicate. Polymerase chain reaction (PCR) was carried out with the use of specific primers for glycosyltransferase gene (forward, GTCCTCTTGGTGACATTTCCCACAC and reverse, TGAGGAAATGCCACCACAGGTACAC). Amplification and detection were performed using LightCycler 2.0 instrument and lightcycler software version 4.0 (Roche, Warszawa, Poland).

### Exposure System

Plant material was exposed to 50 Hz 76–88 μT (60–70 A/m) MF for 30 min in the air-conditioned room with the temperature of 19°C. Taking into account the air flow and the layout of exposition with the minimum distance from the plant of several centimeters there was no significant effect of temperature on the experimental results with the applied MF.

The EMF exposure system was composed of two Helmholtz coils with the inside diameter of 400 mm, external diameter of 462 mm, and 40-mm width with 216 mm spacing. Each of the Helmholtz coils was made of copper wire (2.15 mm in diameter) coiled for 223 winding turns. Both coils were positioned vertically to ensure that the magnetic flux was generated in the horizontal plane. The output of the autotransformer (ZWE Eltra, Bydgoszcz, Poland) connected to the electric energy supply was an electric source for a sinusoidal 50-Hz alternating current (sinusoidal 50-Hz MF) in the experiment. The difference of the potential applied to the coils could be regulated. The amplitude of the current intensity was controlled by the ammeter (Multimeter Fluke 8846A; Fluke, Cleveland, OH, USA). MF strength in the center of the Helmholtz coils can be calculated from the formula derived from Biot–Savart’s law:

$$H_{calculated}=0,7156\cdot \frac{N*I}{r}=0,7156\cdot \frac{223*I}{0,2155}=740\cdot I$$

where: *I – value of current*

r – radius of coils
*N – number of winding turns*.

Higher harmonic waves in the current were monitored during the experiments (Power Quality analyzer Fluke 43, Fluke, Cleveland, OH, USA) and did not exceed 2%. Magnetic induction was measured with EPRI – Emdex II meter (Patterson, CA, USA) and with Holaday HI-3627 meter (Eden Prairie, MN, USA) for traceability. MF strength was kept in our experiments within the range of 60–70 A/m.

Exposure was carried out on potato leaves with 25 mm long and 15 mm width. Orientation of leaf’s stem was parallel to the force lines of MF generated in the exposure system (**Supplementary Figure S1**). During the exposure, the leaves were put on Petri dish (35 mm × 10 mm) positioned in the geometrical center of Helmholtz coils. Additionally, for transgenic plants with 16R promoter, the whole plants in jars were subjected to exposition.

In the case of frequency 50 Hz, the relevant wave length is about 6000 km, therefore the field produced in the exposure system can be considered quasi stationary, i.e., slowly variable in time. It has been proposed that the field produced by alternating 50 Hz current can be described by Biot–Savart formula though it is relevant for direct current (DC). Then, the replacement of alternated current with its root mean square (RMS) value allows for determination of the equivalent strength of MF.

### Statistical Analyses of Experimental Results

*t*-test and confidence intervals (CIs) were calculated using Excel (Microsoft, Warszawa, Poland) to assess statistical significance of the obtained results. *p*-values less than 0.05 were considered statistically significant.

### Searching and Analysis of Potential EMREs in Plant Promoter Regions

Potential defined EMREs, i.e., CTCT sequences (Lin et al., 2001), were searched in 41,036 *S. tuberosum* and 31,036 *A. thaliana* potential promoter sequences, which were defined as regions of 1000 nucleotides upstream of the transcription initiation site of protein-coding nuclear genes; we excluded sequences with unidentified nucleotides in this study. The scanned promoter sequences and gene annotations were obtained from *S. tuberosum* Group Phureja DM1-3 516R44 (CIP801092) genome annotation v3.4 (Xu et al., 2011), deposited in Phytozome v10.2 (Goodstein et al., 2012), and the *Arabidopsis* Information Resource (TAIR) database, release 9 (Lamesch et al., 2012). For each promoter region, the observed number of EMRE sequences was compared with its expected number, which was calculated according to the nucleotide composition of the given promoter sequence. The statistical significance of this comparison was assessed in the test of proportion with the Benjamini-Hochberg multiple comparisons procedure for controlling false discovery rate (Benjamini and Hochberg, 1995) as implemented in R package 3.1.1 (R Development Core Team, 2014). In the putative EMRE search and analysis, in-house written Perl scripts were used.

## Results

In Poland, according to the Regulation of the Minister of Environment of 30 October 2003 (Dz.U. 2003, Nr 192, poz. 1883), 60 A/m is the upper limit intensity for unlimited exposure of humans to 50-Hz MFs. The nCTCTn motif may be responsible for the regulation of gene expression in response to 50-Hz MFs in animal cells (Lin et al., 2001). Therefore, we decided to investigate the potential influence of MFs on plant gene expression involving the EMRE motif.

### Frequency and Distribution of Potential EMREs in Plant Promoter Regions

**FIGURE 1:**
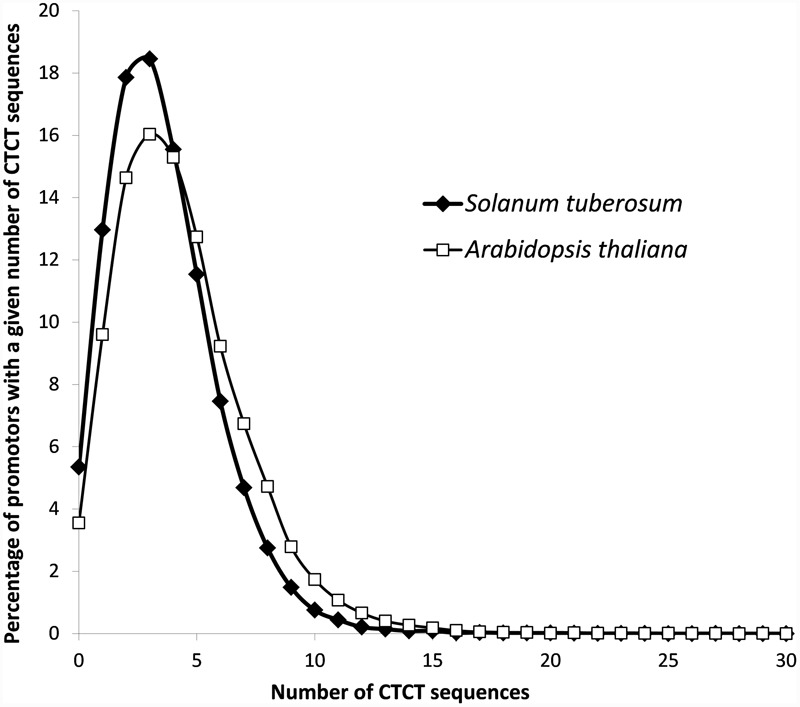
**Distribution of CTCT sequences in the set of potential *S. tuberosum* and *Arabidopsis thaliana* promoters (1000 nucleotides upstream of the transcription start site)**.

To analyze the frequency and distribution of the potential EMRE motif within gene promoters in plants, we included two plant representatives, *S. tuberosum*, the subject of the experimental studies in this paper, and a popular model plant organism, *A. thaliana*. The genome of *S. tuberosum* has the total length of ∼800 Mb that is arranged into 12 chromosomes (Xu et al., 2011), whereas *A. thaliana* has the ∼135 Mb genome that is organized into five chromosomes (Lamesch et al., 2012). We searched potential 1000 bp-promoter sequences from these two genomes. The analysis identified 148,487 in *S. tuberosum* and 134,361 in *A. thaliana* putative EMRE motifs, which gives respective averages of 3.6 and 4.3 such sequences per promoter. In both plants, promoters that contained three CTCT sequences were most abundant (**Figure 1**). They constituted more than 18% (7573) in *S. tuberosum* and 16% (4977) in *A. thaliana* of cases. More than 15% of the promoters (i.e., 6380 in *S. tuberosum* and 4745 *A. thaliana* cases) included four potential EMRE sequences, whereas 10 or more motifs were present in 783 *S. tuberosum* and 1449 and *A. thaliana* promoter regions (**Figure 1**). The motifs were absent from only 2195 and 1103 promoter regions, respectively.

**FIGURE 2:**
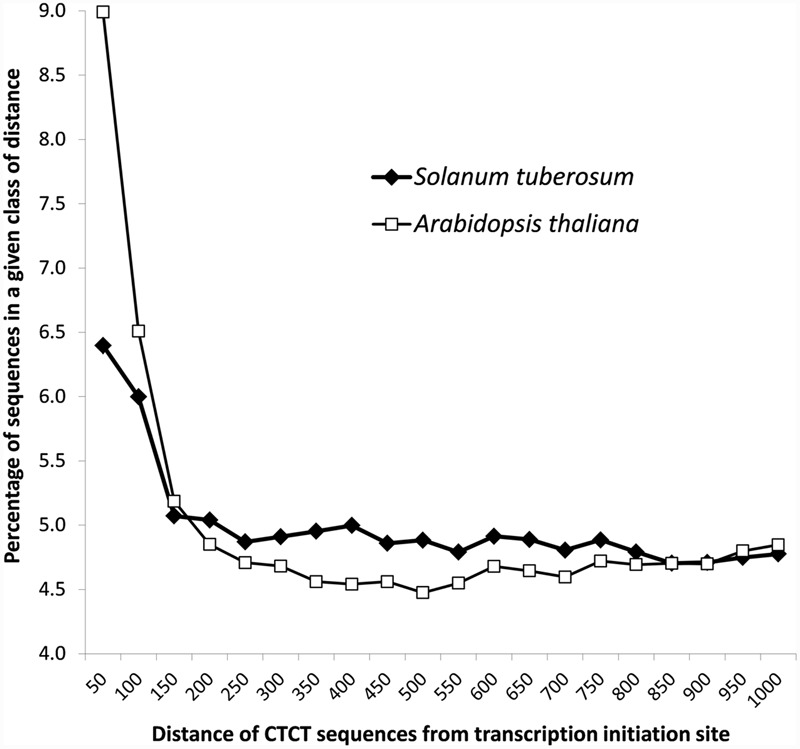
**Distribution of the distance of CTCT sequences from the transcription start site in *S. tuberosum* and *A. thaliana***.

Detailed analyses revealed that CTCT sequences were not randomly distributed in the promoter regions, but rather located close to the transcription initiation site (**Figure 2**). Almost 6.5% and 9% of these sequences (9,500 from *S. tuberosum* and 12,080 *A. thaliana*) were found in less than 50 nucleotides from the transcription start site, whereas more than 12% and 15% of these sequences (18,407 and 20,825 cases) were present in 100 nucleotides upstream of the site, respectively. The contribution of these motifs decreases rapidly at 150 bp from the start transcription site (**Figure 2**).

**FIGURE 3:**
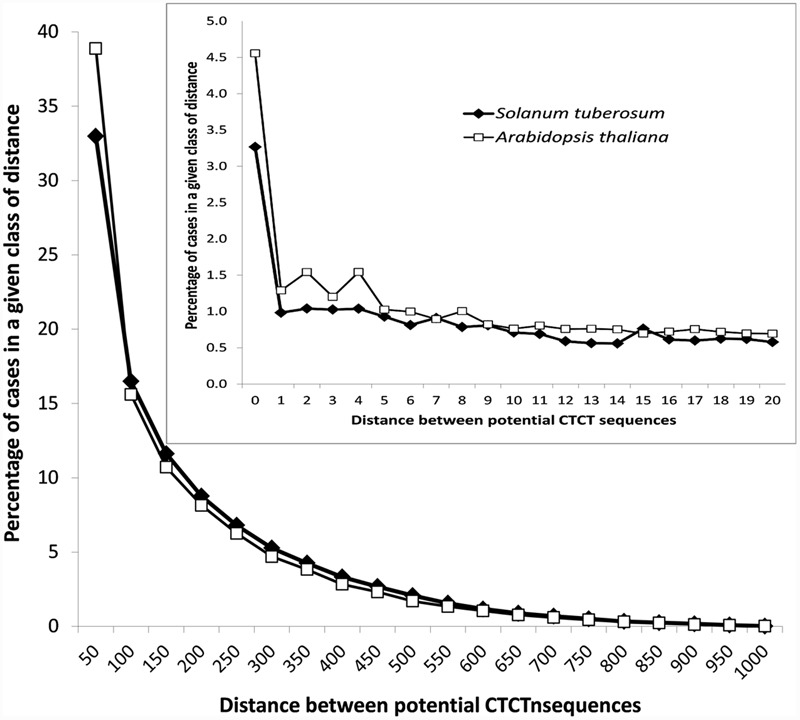
**Distribution of the distances between CTCT sequences found in promoter regions of *S. tuberosum* and *A. thaliana*.** The inset shows the distribution in the narrower range from 0 to 20 nucleotides.

**FIGURE 4:**
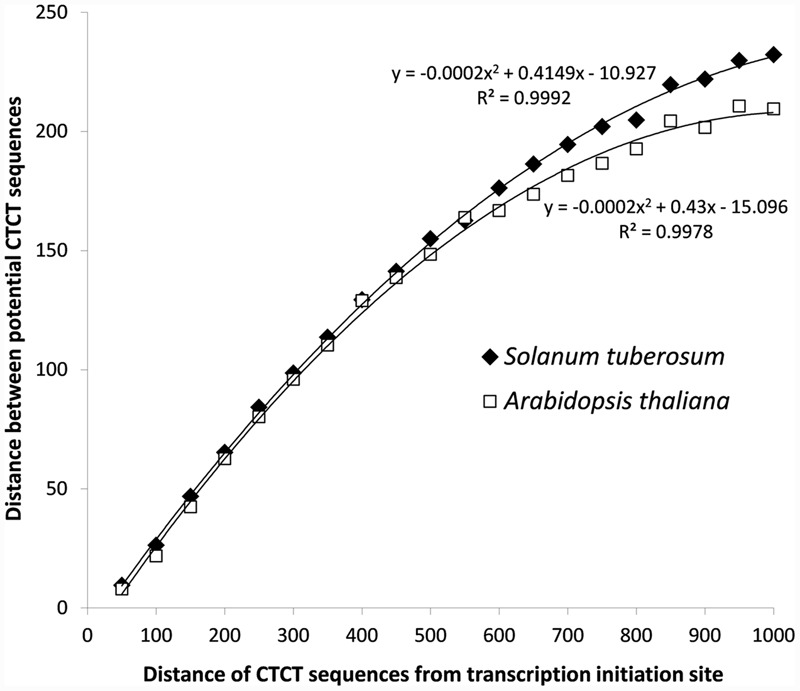
**Relationship of the distance between potential CTCT sequences with their distance from the transcription initiation site for *S. tuberosum* and *A. thaliana***.

In addition to that, the analysis of distances between potential EMRE sequences that were found in promoter regions indicated that many of these sequences are closely packed (**Figure 3**). The average distance between these motifs is 162 and 146 bp for *S. tuberosum* and *A. thaliana*, whereas between these motifs randomly distributed across the promoters much larger, i.e., 271 and 226 bp, respectively. In 36,165 and 40,610 cases (33 and 39%) in *S. tuberosum* and *A. thaliana*, the distance was less than 50 nucleotides, whereas in 3582 and 4757 cases (3.3 and 4.5%), respectively, the sequences were adjacent to each other. If we randomized position of these motifs within their promoter regions, only 0.11% and 0.15% of distances between these motifs were shorter than 50 bp for *S. tuberosum* and *A. thaliana*, and none sequences were adjacent. About 19% and 23% distances up to 20 bp were found for the *Solanum* and *Arabidopsis* genomes, whereas 0.04% and 0.01% for the corresponding randomized data (**Figure 3**, inset). Interestingly, the densely packed CTCT motifs accumulated close to the site of transcription initiation. A clear positive but non-linear relationship can be observed between the distance of potential EMRE sequences and the distance of these sequences from the transcription initiation site (**Figure 4**); the closer the motifs to the transcription initiation site, the smaller distances between these motifs in the studied plants.

To check if the potential EMRE motifs occurred in these promoters by chance, we calculated the expected number of these motifs for each promoter region based on the nucleotide composition of the given promoter sequence and compared the expected number with the observed number of CTCT sequences in this promoter. These sequences occurred four times more often than expected in 126 *S. tuberosum* and 159 *A. thaliana* promoters and three times more often than expected in 728 *S. tuberosum* and 975 *A. thaliana* promoters. However, the excess of the found motifs was statistically significant, with a nominal *p*-value < 0.05, only in 131 for potato and 236 thale cress promoters. When the Benjamini-Hochberg correction for multiple testing was applied, the difference was statistically significant, with an adjusted *p*-value < 0.05 only in 12 potato and four thale cress cases.

The top genes with the largest (>20) number of potential EMRE motifs found in their promoters are listed in **Tables 1** and **2**. If the MF response is positively correlated with the number of these motifs, then the expression of these genes would be potentially regulated by MFs. In *S. tuberosum*, there are some genes with extremely large numbers of the motifs but their function is unknown, whereas in the case of *A. thaliana*, more than one-third of these genes constitute transposable elements. The promoter for the potato gene encoding RHO-related protein is also rich in CTCT sequences. Such proteins transmit a variety of extracellular and intracellular signals by regulating downstream pathways and signaling cascades. These proteins are involved in diverse cellular processes, such as cytoskeletal organization, pollen and vegetative cell growth, hormone responses, stress responses, and pathogen resistance. Moreover, the motif-rich genes also encode putative transcription factors, proteins interacting with DNA, protein kinases and phosphatases, as well as others, which could regulate the expression of other genes and coded proteins under the influence of MFs. Both plants also express pentatricopeptide (PPR) repeat-containing proteins, which are expressed in mitochondria and plastids. In these organelles, the PPR repeat-containing proteins bind organellar transcripts and influence their expression by RNA editing, turnover, processing, or translation (Barkan and Small, 2014); consequently, they have profound effects on organelle biogenesis and function, including photosynthesis, respiration, plant development, and environmental responses.

**Table 1:** Top *Solanum tuberosum* genes with the largest number of CTCT motifs found in their promoter regions.

Motifs’ number	O/E	Nominal *p*-value	Adjusted *p*-value	Locus identifier PGSC0003DMG	Gene model description
195	4.8	2.7×10^-26^	1.1×10^-21^	402023823	Conserved gene of unknown function
162	5.6	1.1×10^-23^	2.2×10^-19^	400036968	Conserved gene of unknown function
147	6.0	3.4×10^-22^	4.7×10^-18^	400008659	RHO-related protein from plants 9 (ROP)
126	6.2	2.8×10^-19^	2.9×10^-15^	400036183	Conserved gene of unknown function
94	6.0	3.4×10^-14^	2.8×10^-10^	400006442	Chlorophyll A-B binding family protein; early light inducible
75	3.7	1.6×10^-08^	8.3×10^-05^	400011588	RING/FYVE/PHD zinc finger superfamily protein; inhibitor of growth
69	3.6	1.1×10^-07^	0.0004	400013655	Zinc finger (CCCH-type/C3HC4-type RING finger) family protein, 113A
65	3.9	9.3×10^-08^	0.0003	400042564	Gene of unknown function
59	6.7	1.2×10^-09^	7.3×10^-06^	400032782	S-domain-2 5; S-receptor kinase
57	8.1	4.8×10^-10^	3.3×10^-06^	400029702	Telomerase activating protein Est1; Smg-7
52	5.5	7.7×10^-08^	0.0003	400006299	Gene of unknown function
42	9.9	4.5×10^-08^	0.0002	400004251	Stress responsive alpha-beta barrel domain protein
27	2.7	0.0074	1.0000	400009231	Transducin/WD40 repeat-like superfamily protein; eukaryotic translation initiation factor 3 subunit
24	3.1	0.0065	1.0000	400027515	Rhodanese/cell cycle control phosphatase superfamily protein; Cdc25
24	2.1	0.0485	1.0000	400030089	Heat-shock protein 70T-2; 70kD
23	3.4	0.0046	1.0000	400012860	Extracellular ligand-gated ion channel
23	3.1	0.0074	1.0000	400001100	Electron transfer flavoprotein alpha; oxidoreductase
23	3.0	0.0087	1.0000	400025959	Pentatricopeptide (PPR) repeat-containing protein
22	4.2	0.0024	1.0000	400013826	Early flowering 3
21	3.3	0.0083	1.0000	400041921	Gene of unknown function
21	3.2	0.0093	1.0000	400030537	60S ribosomal protein L31e family protein
21	3.1	0.0122	1.0000	400035125	Gene of unknown function
21	2.9	0.0148	1.0000	400044565	Gene of unknown function
21	2.6	0.0244	1.0000	400023145	Pentatricopeptide (PPR) repeat-containing protein
21	2.6	0.0256	1.0000	400029893	Gene of unknown function

*O/E is the ratio of the observed to the expected number of motifs in their promoters.*

**Table 2:** Top *A. thaliana* genes with the largest number of CTCT motifs found in their promoter regions.

Motifs’ number	O/E	Nominal *p*-value	Adjusted *p*-value	Locus identifier	Gene model description
50	5.6	1.1×10^-7^	0.0035	AT2G30740	Serine/threonine protein kinase
43	6.1	5.4×10^-7^	0.0083	AT3G31406	Transposable element gene
42	4.7	5.6×10^-6^	0.0438	AT4G04590	Transposable element gene; CACTA-like transposase family
35	5.0	2.5×10^-5^	0.1317	AT2G12510	Transposable element gene; gypsy-like retrotransposon family
33	7.6	4.7×10^-6^	0.0438	AT2G13175	Transposable element gene; CACTA-like transposase family
32	4.7	8.7×10^-5^	0.3872	AT5G28410	Unknown protein
31	3.1	0.0×017	1.0000	AT1G27870	Nucleic acid binding
29	8.2	1.5×10^-5^	0.0913	AT1G33350	Pentatricopeptide (PPR) repeat-containing protein
27	4.1	0.0007	1.0000	AT3G42060	Myosin heavy chain-related
26	6.3	0.0001	0.4803	AT3G47600	Putative transcription factor (MYB94)
25	5.0	0.0005	1.0000	AT3G29610	Transposable element gene
25	4.9	0.0005	1.0000	AT3G55960	NLI interacting factor (NIF) family protein
25	3.5	0.0028	1.0000	AT1G44060	Transposable element gene; CACTA-like transposase family
24	3.5	0.0035	1.0000	AT3G13140	Hydroxyproline-rich glycoprotein family protein
23	5.6	0.0005	1.0000	AT1G63480	DNA-binding family protein
23	5.0	0.0009	1.0000	AT5G58550	Paralog of ETO1, a negative regulator of ACS5 involved in ethylene biosynthesis pathway
23	4.2	0.0019	1.0000	AT1G36403	Transposable element gene; mutator-like transposase family
23	2.5	0.0256	1.0000	AT2G11620	Unknown protein
22	4.0	0.0029	1.0000	AT3G51390	Zinc finger (DHHC type) family protein
22	3.6	0.0050	1.0000	AT5G35066	Unknown protein
22	2.7	0.0189	1.0000	AT3G30837	Transposable element gene; CACTA-like transposase family
22	2.0	0.0827	1.0000	AT1G10330	Pentatricopeptide (PPR) repeat-containing protein
21	5.5	0.0011	1.0000	AT2G28350	Involved in root cap cell differentiation
21	4.3	0.0028	1.0000	AT1G50620	PHD finger family protein
21	4.2	0.0031	1.0000	AT3G42130	Glycine-rich protein
21	2.9	0.0152	1.0000	AT5G28320	Unknown protein
21	2.5	0.0329	1.0000	AT3G43154	Transposable element gene; pseudogene, hypothetical protein
21	2.2	0.0610	1.0000	AT2G34130	Transposable element gene; CACTA-like transposase family
21	2.1	0.0703	1.0000	AT5G30762	Transposable element gene; pseudogene, hypothetical protein
21	1.6	0.2117	1.0000	AT5G32511	Transposable element gene; pseudogene, hypothetical protein

*O/E is the ratio of the observed to the expected number of motifs in their promoters.*

Interestingly, among the *S. tuberosum* genes with the large numbers of potential EMRE motifs are those that code for stress-responsive alpha-beta barrel domain protein and heat-shock protein 70T-2 (**Table 1**). In fact, human HSP70 promoters containing these motifs respond to MFs (Lin et al., 1998, 1999); similarly, we found at least one CTCT motif in the promoters of almost all genes encoding HSP70 in potato (**Table 3**). More than half of the HSP70 genes have more observed motifs than expected. In the case of *A. thaliana* promoters for HSP70 genes, all contained at least one this motif and almost 90% of them had more observed these motifs than expected (**Table 4**).

**Table 3:** *Solanum tuberosum* HSP70-encoding genes with the number of CTCT motifs found in their promoter regions.

Motifs’ number	O/E	Locus identifier PGSC0003DMG
24	2.1	400030089
12	3.1	402031379
10	2.4	400012254
7	1.6	400015920
5	2.4	400024707
5	1.7	400018544
5	1.5	400028634
5	1.2	401031379
4	1.9	400030089
4	1.7	400000398
4	1.6	400024707
4	1.5	400003246
4	1.1	400003122
3	1.5	400008917
3	1.0	400003246
3	0.9	400014212
3	0.9	400014212
2	0.8	400010677
2	0.8	400024887
2	0.6	400010677
2	0.6	400044451
2	0.5	400011197
1	0.5	400028634
1	0.4	400008698
1	0.3	401031379
0	0.0	400003122

*O/E is the ratio of observed to expected number of motifs in their promoters.*

**Table 4:** *Arabidopsis thaliana* HSP70-encoding genes with the number of CTCT motifs found in their promoter regions.

Motifs’ number	O/E	Locus identifier
11	3.0	AT1G79920
9	2.1	AT4G16660
9	2.6	AT5G02500
8	2.0	AT4G24280
6	1.5	AT3G09440
6	1.5	AT5G09590
5	2.3	AT1G79930
5	1.3	AT1G11660
5	1.8	AT2G32120
5	1.6	AT3G12580
5	1.5	AT4G17750
5	1.3	AT5G02490
4	1.7	AT1G16030
3	1.0	AT4G37910
3	1.7	AT4G32208
2	0.7	AT1G09080
1	0.3	AT5G49910

*O/E is the ratio of observed to expected number of motifs in their promoters.*

To look for the elements that could modulate transcription in response to ELF-MF, we further analyzed the sequences of three potato promoters, namely 16R, 20R, and 5UGT. These promoters are well-studied and validated expression models in plants (Korobczak et al., 2005; Łukaszewicz and Szopa, 2005).

### 14-3-3 Protein 16R Promoter

Within the 16R promoter (972 nucleotides in length), the transcription initiation site is located 89 nucleotides upstream of the translation initiation site. This promoter region contains a typical CCAAT box, located at position -147, but lacks a typical TATA box. Among many others, the following putative transcription factor binding sites were found in this promoter: ARF (Auxin Response Factor), light-regulated element GATA, I-box, GT1, elicitor responsive element (ElRE) regulated upon response to infection (TTGACC), and a frequent motif, AATAGAAAA, present in promoters of genes regulated by sucrose levels. Three CTCT motifs were found, which could potentially regulate gene expression after exposure to MFs.

**FIGURE 5:**
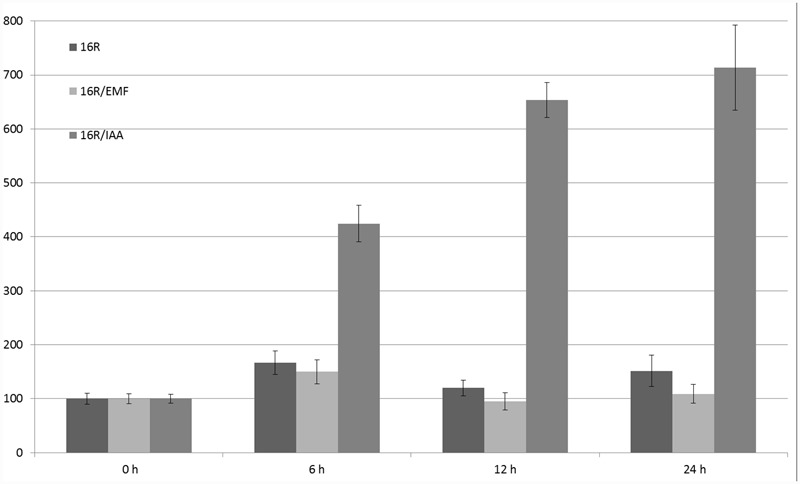
**Regulation of expression of 16R promoter under the influence of extremely low-frequency magnetic field (EMF) and IAA.** The activity of β-glucuronidase (GUS) in the leaves was measured at the start of the experiment (0 h) and after 6, 12, and 24 h. Young leaves of potatoes grown in the greenhouse were cut (in four parts) and incubated in MS medium (control), under MF 61–69 A/m, or with 100 μM IAA. The graph shows the mean values and confidence intervals obtained from 31 replicates. GUS activity in the control was assumed as 100%.

Sucrose and plant hormones (such as IAA, ABA, and salicylic acid) regulated the expression of GUS under control of the 16R promoter (Szopa et al., 2003b). The fastest-acting factor was salicylic acid (2- to 3-fold increase in 6 h after stimulation). The strongest influence on 16R promoter activity was observed in the case of IAA (after 24 h, sevenfold induction), while there was no statistically significant effect of the 50-Hz MF with 60–70 A/m intensity (**Figure 5**).

### 14-3-3 Protein 20R Promoter

The 20R promoter (1239 nucleotides in length) contains a number of motifs that potentially respond to light, amylase boxes, and sequences regulated by ABA and cold (Aksamit et al., 2005). Fewer sequences are potentially responsible for regulation by auxins, salicylic acid, pathogens, sucrose, or ethylene. Within the analyzed promoter, a transcriptional activator involved in flavonoid biosynthesis regulation was also found. Finally, a motif regulated by metals in the mouse gene encoding the murine metallothionein (Koizumi et al., 1999) was identified. Twelve CTCT motifs were also recognized in the promoter.

**FIGURE 6:**
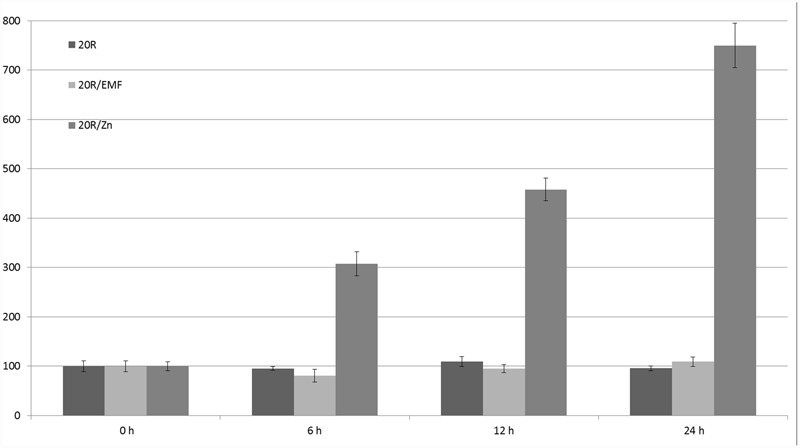
**Regulation of expression of 20R promoter under the influence of EMF and zinc ions (Zn).** GUS activity in the studied leaves was measured at the start of the experiment (0 h) and after 6, 12, and 24 h. Young leaves of potatoes grown in the greenhouse were cut (in four parts) and incubated with 100 μM zinc or exposed to 62–67 A/m MF. The graph represents the mean values and confidence intervals obtained from 34 replicates. GUS activity in the control was assumed as 100%.

**FIGURE 7:**
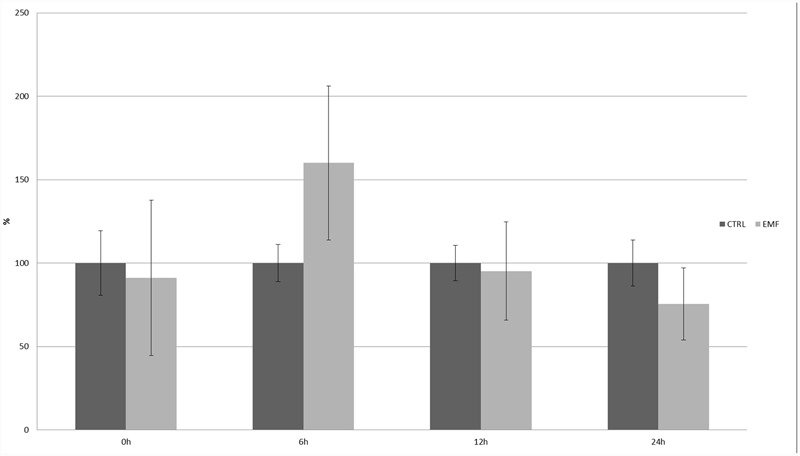
**Expression of 20R promoter under the influence of EMF after 30 min exposition of whole 14 day old potato plants.** GUS activity in the studied leaves was measured at the start of the experiment (0 h) and after 6, 12, and 24 h. The graph represents the mean values and confidence intervals obtained from three replicates. GUS activity at the start of the experiment (0 h) in the control was assumed as 100%.

Different factors were tested using this promoter: ABA, ethylene, auxin, salicylic acid, pathogen infections, metals (cadmium, zinc, and copper), salt, glycol, light, wounding, low temperature, and sucrose. Auxin, ethylene, salicylic acid, pathogen infection, and sucrose did not affect GUS activity (Aksamit et al., 2005); however, ABA, cold, light, and heavy metals did regulate GUS expression. The strongest influence on the 20R promoter’s activity (eightfold induction) was observed in the case of zinc stress after 24 h; the 50-Hz MF had no statistically significant effect on the activity of the 20R promoter (**Figure 6**). Additionally, to exclude impact of mechanical stress an experiment was performed on 14-days whole potato plants (**Figure 7**). In the leaves of plants exposed to EMF, no statistically significant increase in GUS activity was observed 6 h after the exposition. The activity decreased in the following time-points and was similar to the control.

### Glucosyltransferase (5UGT) Promoter

**FIGURE 8:**
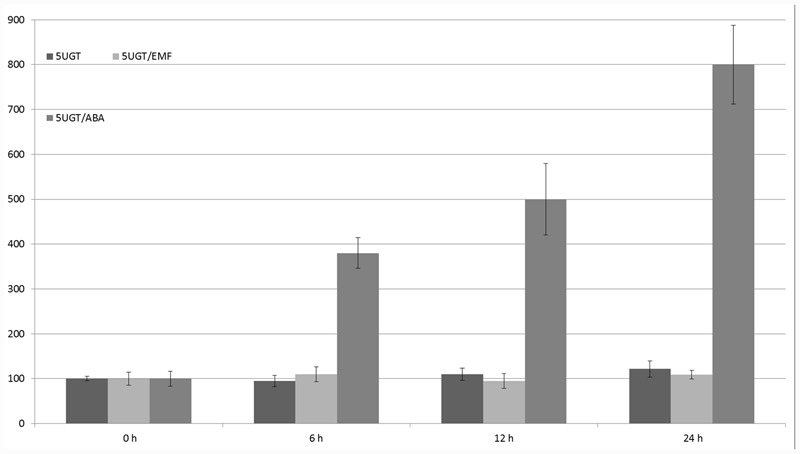
**Regulation of expression of the 5UGT promoter under the influence of EMF and ABA.** GUS activity in the studied leaves was measured at the start of the experiment (0 h) and after 6, 12, and 24 h. Young leaves of potatoes grown in the greenhouse were cut (in four parts) and incubated with 100 μM ABA or 62–67 A/m MF. The graph represents the mean values and confidence intervals obtained from 32 replicates. GUS activity in the control was assumed as 100%.

The glucosyltransferase promoter (1625 nucleotides in length) was isolated from the wild potato *S. sogarandinum*. Within its entire sequence, several motifs were found upstream of the translation start site that are potentially recognized by transcription factors and involved in the regulation of responses to UV light, ABA, light, sucrose, and potentially MF because five CTCT motifs were identified. Using heterologous expression in *S. tuberosum*, the 5UGT promoter was induced by UV light, cold, light, ABA, and salt (NaCl). The effect of cooling and light, as well as ABA and cold, were synergistic. The 5UGT promoter’s activity was inhibited by sugar concentrations above 2% (Lorenc-Kukuła et al., 2004). The 5UGT promoter’s activity increased with time while under the influence of ABA was the highest (eightfold) after 24 h of incubation. However, there was no statistically significant effect of the 50-Hz MF with 60–70 A/m intensity (**Figure 8**). This result was also confirmed in *S. sogarandinum* by mRNA (qPCR) and protein (Western Blot) quantification (**Supplementary Figures S2**, **S3**).

## Discussion

It was postulated that MFs may affect DNA by moving charges (Blank and Goodman, 1999). In agreement with that, the movement of electrons in DNA was observed (Wan et al., 1999; Porath et al., 2000). Moreover, it was suggested that conduction in DNA depends on its structure (Meggers et al., 1998). Therefore, it is reasonable to assume that MFs could interact preferentially with some specific DNA sequences. It was assumed that, three CTCT sequences (EMREs) in the human *hsp70* and *c-myc* promoters regulate expression in response to MF exposure for 30 min at 60 Hz and 8 μT (Lin et al., 1997, 1998, 1999, 2001). Although, such response to magnetic flux density (8 μT = 10 A/m) is below the upper limit (60 A/m) regarded as safe (Zmyslony et al., 2005), these values are frequently measured in households, especially those under overhead power lines (Vulevic and Osmokrovic, 2011). We have previously shown that some transcriptionally active motifs within promoters could be conserved across plant and animal taxa, for example, those induced by metals (Aksamit et al., 2005).

Thus, the purpose of this study was to evaluate the hypothesis that 60 A/m alternating MF may regulate plant gene expression through CTCT motifs. First, we analyzed how often this motif occurred in promoters throughout the genome of *S. tuberosum* and the model plant *A. thaliana*. After examining more than 40,000 in *S. tuberosum* and 30,000 in *A. thaliana* potential promoters and, with lengths of 1000 nucleotides, we found, about 150,000 in *S. tuberosum* and 130,000 in *A. thaliana* putative EMRE motifs appearing within the analyzed promoters, about four times on average. Such a high frequency of potential EMRE motif would suggest that this element alone could not be responsible for the precise regulation of gene expression in response to the given factor (e.g., a 50-Hz MF). This could partially explain previous failures to obtain repeatable effects (Heredia-Rojas et al., 2010; Buchachenko, 2016) and supports the hypothesis that additional elements should assist in changing expression in response to MFs.

Although our searches revealed that the CTCT motifs are widely distributed in the studied plant genomes, the conserved test verifying their presence by chance in promoter regions based on their general nucleotide composition showed only 12 and 4 significant cases for *S. tuberosum* and *A. thaliana*, respectively. However, this statistical procedure tests only the presence CTCT motifs but not their arrangements. Our detailed analyses demonstrated that the CTCT sequences are not randomly distributed within promoter regions but are usually located near the transcription initiation site, where they form clusters, i.e., are laid in a very close distance to each other. The majority of these motifs (about 3582 in *S. tuberosum* and 4757 in *A. thaliana*) are adjoining. The organization of these motifs in clusters may suggest some regulation of gene expression, involving the cooperative binding of transcription factors to DNA. Moreover, it cannot be excluded that some of the neighboring stretches of CTCT were generated by CT dinucleotide repeat expansion, e.g., by DNA polymerase slippage mutations. The distribution of CTCT sequences may be also influenced by genome rearrangements and amplifications mediated by transposable elements (Bennetzen, 2000; Fedoroff, 2000; Feschotte and Pritham, 2007), because these motifs were often found near such elements (**Tables 1** and **2**).

However, the non-random distribution and composition of these motifs suggests that many of them overlap or are placed within so-called GAGA elements, which consist of dinucleotide repeats with the pattern (GA)_n_/(TC)_n_. These elements are a target for specific protein complexes, replacing nucleosomes to create a local chromatin environment, which enables a variety of regulatory responses (Lehmann, 2004). GAGA elements can also be related to the epigenetic regulation of homeotic genes and may influence the promoter-proximal pausing of RNA polymerase II (Lehmann, 2004; Fujioka et al., 2008; Lee et al., 2008; Vaquero et al., 2008). Although the GAGA elements were usually studied in *Drosophila melanogaster* in the context of the regulation of numerous developmental genes (Lehmann, 2004), such motifs and the proteins interacting with them were also characterized in plants (Sangwan and O’Brian, 2002; Santi et al., 2003; Meister et al., 2004; Roig et al., 2004; Kooiker et al., 2005; Wanke et al., 2011). Because the expression pattern of these proteins, designated as BBR/BPC, is widespread and their potential target DNA motifs are also numerous in plant genomes, it seems that these factors may influence the expression of various genes involved in different plant processes, in addition to homeobox genes.

The transcriptional activation by GAGA factors and the presence of GAGA elements in promoter regions were also reported for *hsp70* genes (Granok et al., 1995; Wilkins and Lis, 1997; Georgel, 2005). Interestingly, human HSP70 promoters include CTCT motifs that respond to MFs (Lin et al., 1998, 1999). Our analysis of potential promoters in almost all *S. tuberosum* and all *A. thaliana hsp70* genes also revealed the presence of such motifs, which would suggest that the expression of these genes could be regulated by MFs (**Table 2**). Other genes (e.g., those encoding putative transcription factors, pentatricopeptide repeat-containing proteins, or RHO-related protein) may be also considered MF-sensitive because their promoters contain a significant number of CTCT motifs (**Tables 1** and **2**).

It would be worthwhile to reproduce an easy experimental system to study the impact of MFs on living organisms, including plants. Therefore, in addition to the statistical evaluation of plant promoters, we tested the impact of MFs on stress-related promoters in *S. tuberosum*; however, in the studied experimental conditions, there was no significant regulatory effect of the 50-Hz MF in the range of 60–70 A/m on the expression of 14-3-3 protein gene promoters (16R and 20R) or the glucose 5UGT transferase promoter. Besides the assumption that the EMRE does exist but plants do not respond to MFs because of inappropriate parameters used in the experiments, there are several other possible explanations. One of them is that the CTCT sequences that might act as EMREs should be separated by specific distances. It is also possible that the presence of other motifs and interactions between various transcription factors are necessary for ELF-MF perception, which is very common in many regulators of gene expression. In addition to other uncontrolled parameters that lead to cell line-dependent construct expression, there are clear experimental differences between animal (Jin et al., 1997) and plant (Michelet et al., 1994) cells. In the animal cell experiments, EMF stimuli of less than 1 μT elicited transcripts within 5 min, stress proteins within 20 min, and synthesis gradually decreased after about 3 h. In this work the 30 min EMF stimuli was evaluated after 6, 12, and 24 h. Plants are usually grown in laboratories in temperature about 20°C lower than that in animal cells. Therefore, cellular responses, according to the Arrhenius equation, would be roughly fourfold slower in plant cells incubated in 18°C than in animal cells in 37°C. Consequently, stress responses in plants are often much slower than in animals, with the highest level of reporter proteins occurring often after 24 h (**Figures 5**–**7**). Indeed, in plant cells, the production of proteins on once-produced mRNA template may be constant for at least 6 h, resulting in steady accumulation of a reporter protein. Once produced and accumulated, the reporter protein, such as GUS, is relatively stable and may be detected for several hours after translation. Consequently, even very short transcriptional activation should be visible in our experimental conditions except for very weak activation, resulting in signal lower than background.

The presented results did not confirm the hypothesis that CTCT motifs are EMREs in the studied plant promoters, which seemed promising because they contain CTCT motifs and are known to response to stress conditions. Although we observed no significant response to MF in the plant model, it cannot be excluded that other promoters or motifs can fulfill such function. Moreover, the response can be realized by a complex regulatory network that escapes the simple promoter studies. The response could be initiated by a subset of genes whose promoters can have some MF-specific motifs. Products of these genes could further serve as transcription factors for many other genes whose promoters may not have such specific motifs. These genes may not give positive response to MF if other set of regulated genes are not sufficiently expressed. This complicated network regulation of gene expression requires a separate approach to particular cases and selection of the promoters to GUS/GFP studies without additional knowledge would be misleading. On the other hand, widespread criticism of the original paper about the presence of EMREs, suggests that such elements do not exists in plants. Therefore, the analysis of promoter regions at the specific genes requires a special and separate consideration.

## Author Contributions

MŁ, DS: study concept and design, data acquisition, statistical analysis, analysis and interpretation, manuscript drafting, editing and revision, manuscript final version approval, PM: data acquisition, statistical and bioinformatic analyses, analysis and interpretation of results, manuscript drafting, editing and revision, manuscript final version approval, AA-S: data acquisition, analysis and interpretation, manuscript final version approval, KK: data acquisition, analysis, and interpretation.

## Conflict of Interest Statement

The authors declare that the research was conducted in the absence of any commercial or financial relationships that could be construed as a potential conflict of interest.

## Supplementary Material

FIGURE S1 **Exposure system from the top (A)** and side **(B)**. Orientation of leaf’s stem against the force lines of magnetic field generated in exposure system. C1, C2, coils; L, magnetic field exposed leaf; P-base, structural elements supporting Helmholtz coils; W, force lines of magnetic field; S-Petri dish with the exposed sample.

FIGURE S2 **Western analysis of proteins isolated from leaves of control and treated *S. sogarandinum* plants.** 80 μg of protein was applied onto each slot of SDS-polyacrylamide gel electrophoresis and the blot was probed with antibody anti-recombinant GT protein. The treatment conditions are marked over the result of western analysis. C1 and C2- control (untreated sample), samples with 30-min treatment in the magnetic field 62–67 A/m and collected after 6 h (6/1, 6/2, 6/3, 6/4), 12 h (12/1, 12/2, 12/3, 12/4), and 24 h (24/1, 24/2, 24/3, 24/4).

FIGURE S3 **Quantification of GT gene expression by real time PCR in *S. sogaradinum* in response to MF exposition after 0, 6, 12, and 24 h**.
