# Supplementary material for: Electromagnetic Field Seems to Not Influence Transcription via CTCT Motif in Three Plant Promoters
Source: Front Plant Sci. 2017 Mar 7;8:178. doi: 10.3389/fpls.2017.00178 (PMC5339303; doi:10.3389/fpls.2017.00178)
Supplement: FIGURE S1 — Exposure system from the top (A) and side (B). Orientation of leaf’s stem against the force lines of magnetic field generated in exposure system. C1, C2, coils; L, magnetic field exposed leaf; P-base, structural elements supporting Helmholtz coils; W, force lines of magnetic field; S-Petri dish with the exposed sample. [file Data_Sheet_1.docx]

**A**

P

L

W

S

216 mm

**B**

C2

C1

L

P

W

S

**S1 . Exposure system from the top (A) and side (B).**

Orientation of leaf’s stem against the force lines of magnetic field generated in exposure system

C1, C2 - Coils

L -magnetic field exposed leaf

P-base, structural elements supporting Helmholtz coils

W- force lines of magnetic field

S-Petri dish with the exposed sample.
