# Supplementary figures and images for: Electromagnetic Field Seems to Not Influence Transcription via CTCT Motif in Three Plant Promoters
Source: Front Plant Sci. 2017 Mar 7;8:178. doi: 10.3389/fpls.2017.00178 (PMC5339303; doi:10.3389/fpls.2017.00178)

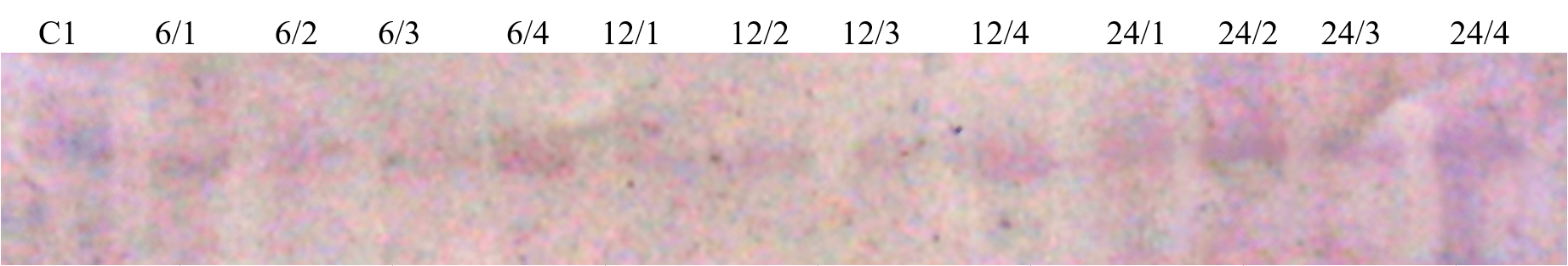

Supplement: FIGURE S2 — Western analysis of proteins isolated from leaves of control and treated S. sogarandinum plants. 80 μg of protein was applied onto each slot of SDS-polyacrylamide gel electrophoresis and the blot was probed with antibody anti-recombinant GT protein. The treatment conditions are marked over the result of western analysis. C1 and C2- control (untreated sample), samples with 30-min treatment in the magnetic field 62–67 A/m and collected after 6 h (6/1, 6/2, 6/3, 6/4), 12 h (12/1, 12/2, 12/3, 12/4), and 24 h (24/1, 24/2, 24/3, 24/4). [file Image_2.PNG]

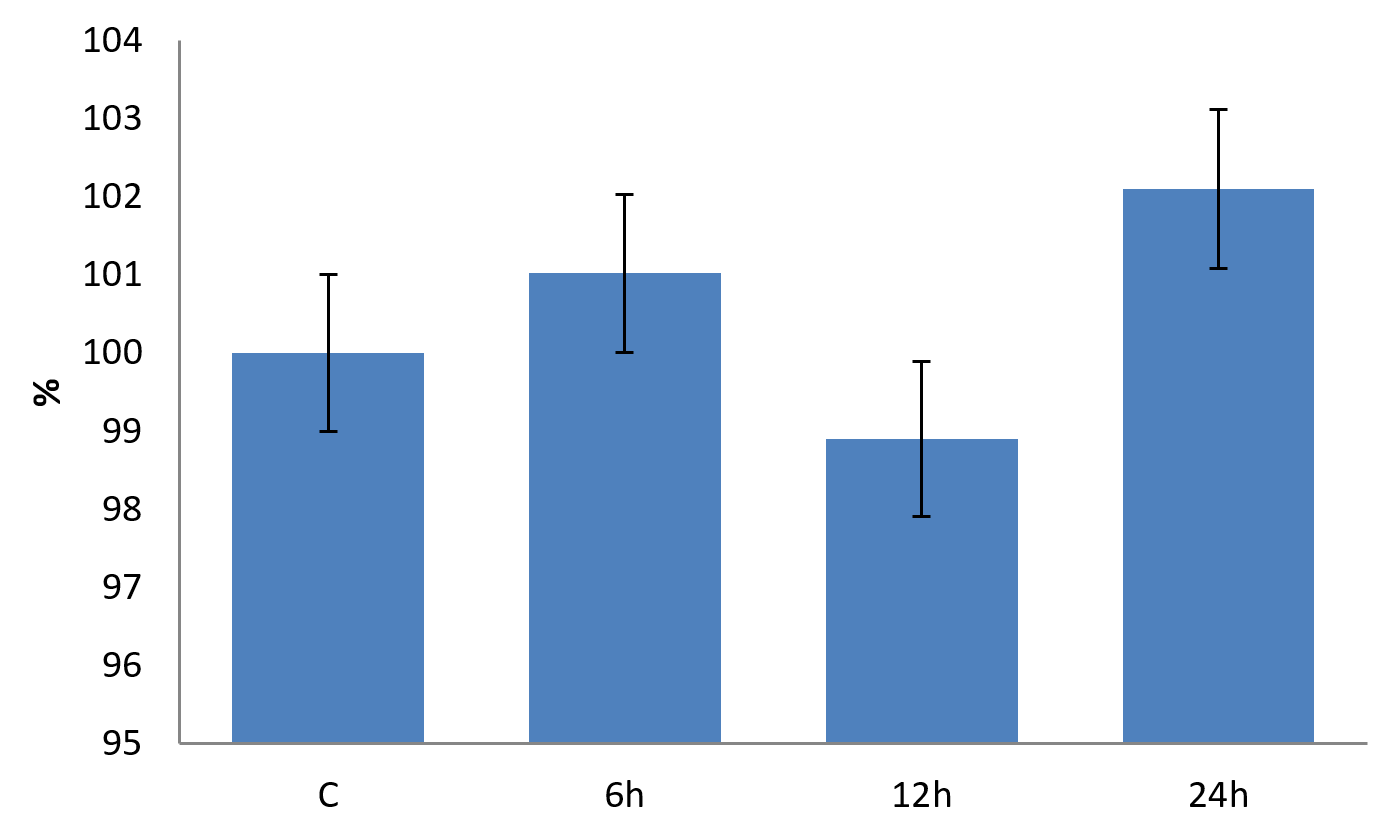

Supplement: FIGURE S3 — Quantification of GT gene expression by real time PCR in S. sogaradinum in response to MF exposition after 0, 6, 12, and 24 h. [file Image_3.TIF]
